# Perforation of Anterior Mitral Valve Leaflet Aneurysm: Complication of Enterococcus Faecalis Infective Endocarditis

**DOI:** 10.7759/cureus.10249

**Published:** 2020-09-04

**Authors:** Anuradha Kolluru, Subrat Behera, Vijay Damarla, Venkat Rajasurya

**Affiliations:** 1 Cardiology, Decatur Memorial Hospital, Decatur, USA; 2 Family Medicine, Mercyone Siouxland Medical Center, Sioux City, USA; 3 Hematology and Oncology, Decatur Memorial Hospital, Decatur, USA; 4 Pulmonary Critical Care, Novant Health, Winston-Salem, USA

**Keywords:** infective endocarditis, mitral leaflet aneurysm

## Abstract

Infective endocarditis (IE) is a potentially fatal disease if untreated and transesophageal echocardiogram should be performed in all suspected cases. We report a complicated case of infective (Enterococcal faecalis) endocarditis in an elderly man who recently had a genitourinary procedure. He presented with decompensated congestive heart failure due to valvular insufficiency and was found to have multiple vegetations on aortic and mitral valves with aneurysm and perforation of the anterior mitral valve leaflet. He was appropriately treated with antibiotics and surgery. Echocardiography plays central role in risk stratification, evaluation, diagnosis and management.

## Introduction

In the United States, the mean age of infective endocarditis has risen, and currently, more than 50% of patients are older than 50 years. Infective endocarditis (IE) is three times as common in males as in females. It has no racial predilection. The mortality rate within one year of acquiring infection is almost 30% [1]. IE remains a diagnostic and therapeutic challenge.

## Case presentation

A 73-year-old male presented with symptoms of worsening shortness of breath on exertion, generalized tiredness, subjective low-grade fever, fatigue, orthopnea, and paroxysmal nocturnal dyspnea for a couple of weeks. His only relevant past medical history was transurethral resection of localized bladder tumor a couple of months ago with no other systemic therapy. He maintained an active and healthy lifestyle, no alcohol, smoking, or illicit drug abuse. His initial vitals were stable except tachycardia. Pertinent physical examination findings were bilateral rales in lung bases, holosystolic murmur at the apex, and mild pedal edema. Electrocardiogram showed sinus tachycardia, and chest X-ray findings were consistent with congestive heart failure. Initial blood work revealed electrolytes in the normal range, leukocytosis, elevated erythrocyte sedimentation rate (ESR) and C-reactive protein (CRP).

He was hospitalized and was started on antibiotics as his blood cultures were positive for Enterococcus faecalis. Transthoracic echocardiography was limited to visualize vegetations but suggested moderate to severe regurgitation of both aortic and mitral valves with preserved left ventricular ejection fraction. Within 48 hours of admission, his oxygen requirements increased in spite of diuresis, and hence urgent transesophageal echocardiogram (TEE) was done to evaluate for valvular insufficiency. TEE (Videos 1-2) showed aortic regurgitation, multiple vegetations on aortic valve (all three cusps) with one prolapsing into the left ventricular outflow tract, aneurysm of anterior mitral valve leaflet with perforation and severe mitral regurgitation. Tricuspid and pulmonary valves were unremarkable.

**Video 1:** Aneurysm of the anterior mitral valve leaflet, multiple vegetations on aortic and mitral valve noted on transesophageal echocardiogram

**Video 2:** Transesophageal echocardiography depicting color turbulence due to perforated anterior mitral leaflet aneurysm

He was diagnosed as healthcare-associated Enterococcus faecalis infective endocarditis complicated with mitral valve aneurysm and perforation. Although he did receive intravenous (IV) antibiotic prophylaxis preoperatively for his transurethral resection of bladder tumor procedure but was complicated with urinary tract infection, which was not adequately treated due to his noncompliance.

He was transferred to tertiary care for valve surgery due to decompensated congestive heart failure. Intraoperatively multiple vegetations on aortic cusps, aneurysm, and perforation of the anterior mitral valve were confirmed with no aortic root abscess. He underwent resection of the anterior mitral leaflet, preservation of posterior leaflet, preservation of sub valvar apparatus, replacement of both aortic and mitral valves successfully with minimal post-operative complications. He was discharged on antibiotics (vancomycin and gentamicin) in stable condition. He was followed in the clinic after six months and had been doing well and completed his antibiotics course and has returned to work.

## Discussion

Infective endocarditis is a potentially life-threatening disease if not treated timely. Identifying early surgical indications is very important to improve survival [2]. There has been a change in the epidemiology of infective endocarditis in developed countries. Healthcare-associated infective endocarditis accounts for more than 25% cases and seen in the elderly population with no known prior cardiac history. Healthcare-associated IE is an emerging facet of the disease, which is a source of concern because of its frequency and severity [3]. Enterococcus faecalis endocarditis is associated with genitourinary procedures. To minimize the risk of bacteremia in healthcare facilities, significant efforts are undertaken as healthcare-associated IE may be viewed as an undesirable effect of healthcare universalization [4].

In the era of intravascular devices, Staphylococcus aureus bloodstream infections have become the primary pathogen of endocarditis. After Streptococci viridans and Staphylococci, the third leading cause of bacterial endocarditis is Enterococcus. It approximately accounts for eight percent of all cases of bacterial endocarditis [5]. Elderly men subjected to multiple genitourinary procedures, intravenous drug users, women in postpartum and with genitourinary infections are at the highest risk for enterococcal endocarditis [6].

Mitral valve aneurysm is a rare entity with the incidence reported as only 0.2-0.29% [7]. It is a saccular outpouching of the mitral leaflet into the left atrium. It expands on systole and collapses in diastole. The anterior leaflet is more commonly involved than the posterior leaflet [8]. The exact mechanism for its development is not known. A weakening of the mitral leaflet can be due to connective tissue diseases or from increased pressure from the left ventricle (in endocarditis or rheumatic disease). 

So far, to our best knowledge, only 11 cases of enterococcal endocarditis causing mitral valve aneurysm have been reported by systematic literature search (Medline (via PubMed), Embase, Scopus, and Google Scholar). The perforation of mitral valve aneurysm due to Enterococcus faecalis endocarditis is rare, and only six identified case reports are available in the literature. Mitral valve aneurysm is typically seen in the presence of aortic valve endocarditis. In the setting of aortic valve endocarditis, the physical trauma and occult infection incurred by infected aortic regurgitant jet to anterior mitral leaflet are thought to be the etiology of development of mitral valve aneurysm. Direct extension of infection to mitral-aortic intervalvular fibrosa can also result in aortic abscess and mitral aneurysm formation. This valvulitis leads to weakening and saccular outpouching, evidenced as scar and granulation tissue on microscopic examination.

Perforation or rupture is the most dreaded complication of mitral valve aneurysm, as it results in rapid hemodynamic deterioration leading to acute pulmonary edema from acute severe mitral regurgitation. Hence the awareness of this rare entity will lead to timely diagnosis and treatment, which can help prevent this catastrophic complication [9, 10]. Cardiothoracic surgery is indicated for large aneurysms and perforated aneurysms. Conservative treatment can be an alternative option for small uncomplicated aneurysms but needs close follow up and monitoring. 

This case highlights the role of TEE in helping the diagnosis of infective endocarditis and its complications early on. Mitral valve aneurysm is one of the rare but catastrophic complications.

## Conclusions

We present a rare but fatal complication of infective endocarditis, the mitral valve aneurysm and emphasize the role of TEE in early diagnosis and surgical referral. TEE was critical in making an accurate diagnosis leading to timely intervention and improved outcome.

## References

[1] Cahill TJ, Baddour LM, Habib G (2017). Challenges in infective endocarditis. J Am Coll Cardiol.

[2] O’Gara PT (2007). Infective endocarditis 2006: indications for surgery. Trans Am Clin Climatol Assoc.

[3] Selton-Suty C, Célard M, Le Moing V (2012). Preeminence of Staphylococcus aureus in infective endocarditis: a 1-year population-based survey. Clin Infect Dis.

[4] Lomas JM,  Martínez-Marcos FJ,  Plata A (2010). Healthcare-associated infective endocarditis: an undesirable effect of healthcare universalization. Clin Microbiol Infect.

[5] Fabri J, Issa VS, Pomerantzeff PM, Grinberg M, Barretto AC, Mansur AJ (2006). Time-related distribution, risk factors and prognostic influence of embolism in patients with left-sided infective endocarditis. Int J Cardiol.

[6] Miro JM, Moreno A, Mestres CA (2003). Infective endocarditis in intravenous drug abusers. Curr Infect Dis Rep.

[7] Vilacosta I, Román J, Sarriá C (1999). Clinical, anatomic, and echocardiographic characteristics of aneurysms of the mitral valve. Am J Cardiol.

[8] Işılak Z, Uzun M, Yalçın M, Kılıçarslan F (2013). An adult patient with the ruptured aneurysm of mitral valve posterior leaflet. Anadolu Kardiyol Derg.

[9] Reid CL, Chandraratna AN, Harrison E (1983). Mitral valve aneurysm: clinical features, echocardiographic-pathologic correlations. J Am Coll Cardiol.

[10] Mollod M, Felner KJ, Felner JM (1997). Mitral and tricuspid valve aneurysms evaluated by transesophageal echocardiography. Am J Cardiol.

